# Edoxaban Dose Adjustment and Age-Associated Outcomes in Patients With Atrial Fibrillation Post-Transcatheter Aortic Valve Replacement

**DOI:** 10.1016/j.jacadv.2025.102329

**Published:** 2025-11-19

**Authors:** Nicolas M. Van Mieghem, Cathy Chen, Christian Hengstenberg, Johanna Van Zyl, Tetsuya Kimura, Irene Lang, Roxana Mehran, Johny Nicolas, Martin Unverdorben, J.L. Zamorano, George D. Dangas

**Affiliations:** a Department of Cardiology, Cardiovascular Institute, Thoraxcenter, Erasmus University Medical Center, Rotterdam, the Netherlands; b Global Medical Affairs, Daiichi Sankyo, Inc., Basking Ridge, New Jersey, USA; c Division of Cardiology, Department of Internal Medicine II, Vienna General Hospital, Medical University, Vienna, Austria; d Department of Medical Science, Daiichi Sankyo Co., Ltd., Tokyo, Japan; e Zena and Michael A. Wiener Cardiovascular Institute, Mount Sinai Hospital, New York, New York, USA; f Icahn School of Medicine, Mount Sinai Hospital, New York, New York, USA; g Department of Cardiology, University Hospital Ramón y Cajal, Madrid, Spain; h National and Kapodistrian University of Athens, School of Medicine, Athens, Greece

**Keywords:** atrial fibrillation, dose adjustment criteria, edoxaban, major bleeding, transcatheter aortic valve implantation

## Abstract

**Background:**

In ENVISAGE-TAVI AF (EdoxabaN Versus standard of care and theIr effectS on clinical outcomes in pAtients havinG undergonE Transcatheter Aortic Valve Implantation-in Atrial Fibrillation), there were more bleeding events with edoxaban 60 mg than vitamin K antagonists (VKAs) in patients with atrial fibrillation (AF) after transcatheter aortic valve replacement (TAVR).

**Objectives:**

This analysis evaluated the impact of edoxaban dose adjustment criteria (eDAC) by age and treatment group (edoxaban vs VKA) on clinical events in patients with AF post-TAVR.

**Methods:**

In this ENVISAGE-TAVI AF on-treatment analysis, patients received edoxaban 60 mg once daily—adjusted to 30 mg if they met ≥1 eDAC (creatinine clearance 15 to ≤50 mL/min, body weight ≤60 kg, or concomitant use of potent P-glycoprotein inhibitors)—or VKA. Clinical outcomes were compared between patients with vs without eDAC by age (<80 vs ≥ 80 years) and by treatment group.

**Results:**

Of 1,377 patients, 637 (46%) met eDAC; 740 (54%) did not. Patients with vs without eDAC had significantly higher rates of cardiovascular death (HR: 1.73; 95% CI: 1.01-2.95; *P* = 0.045). Patients aged ≥80 years without eDAC experienced higher annualized major bleeding (HR: 1.86; 95% CI: 1.04-3.32) and major gastrointestinal bleeding (MGIB) (HR: 3.79; 95% CI: 1.56-9.25) rates with edoxaban vs VKA. Rates of MGIB almost doubled in edoxaban-treated patients without vs with eDAC (8.03%/year vs 4.65%/year). A similar effect was seen in patients aged <80 years without vs with eDAC (4.22%/year vs 2.98%/year).

**Conclusions:**

Patients aged ≥80 years without eDAC were at a higher risk of major bleeding and MGIB events with edoxaban 60 mg vs VKA. An optimized edoxaban dose for octogenarians with AF post-TAVR, regardless of eDAC, may help improve outcomes. (Edoxaban Compared to Standard Care After Heart Valve Replacement Using a Catheter in Patients With Atrial Fibrillation [ENVISAGE-TAVI AF]; NCT02943785)

Transcatheter aortic valve replacement (TAVR) is the preferred treatment option for aortic stenosis in selected patients with symptomatic severe aortic stenosis.^1^^,^^2^ Atrial fibrillation (AF) occurs frequently in patients with aortic stenosis, and the recommended treatments to prevent thromboembolic events include direct oral anticoagulants (DOACs) or vitamin K antagonists (VKAs).^2^ However, patients aged ≥80 years with AF after TAVR who take the recommended full dose of DOACs to prevent thromboembolic events have a higher risk of bleeding compared with younger patients on the same dose.^3^^,^^4^ A randomized controlled trial in older patients with AF living with frailty (aged ≥75 years with a Groningen frailty indicator ≥3) also found more bleeding events when these patients were switched from VKA to full-dose DOAC.^5^ Conversely, low utilization and nonrecommended dosing of DOACs may lead to a higher risk of ischemic events.^6^, ^7^, ^8^, ^9^ Although patients aged ≥80 years were over-represented in the ENVISAGE-TAVI AF (EdoxabaN Versus standard of care and theIr effectS on clinical outcomes in pAtients havinG undergonE Transcatheter Aortic Valve Implantation-in Atrial Fibrillation) trial,^10^ they are often under-represented in randomized controlled trials. As such, there are limited randomized controlled trial data on the efficacy and safety of lower doses of DOACs in patients aged ≥80 years.

In the ENVISAGE-TAVI AF trial (NCT02943785), edoxaban was associated with more major bleeding (MB) and major gastrointestinal bleeding (MGIB) in patients on edoxaban 60 mg compared with VKA.^10^ Previous analyses suggest that older patients on DOACs vs VKA have similar rates of ischemic stroke or systemic embolic events and lower rates of MB events when given the 30-mg dose.^11^^,^^12^ For example, a prespecified analysis of the pivotal ENGAGE AF-TIMI (Effective aNticoaGulation with factor Xa next GEneration in Atrial Fibrillation–Thrombolysis In Myocardial Infarction study) 48 trial—a study which looked at patients with nonvalvular AF treated with edoxaban vs VKA—demonstrated that patients aged ≥75 years had a significantly lower MB event rate with edoxaban 30 mg vs warfarin.^11^ These findings suggest that edoxaban 30 mg may represent an optimized dosage for older patients. The aims of this post hoc analysis of the ENVISAGE-TAVI AF trial were: 1) to assess differences in clinical outcomes in patients with vs without edoxaban dose adjustment criteria (eDAC); and 2) to determine if age (<80 years vs ≥80 years) or oral anticoagulant treatment (edoxaban vs VKA) differentially impact clinical outcomes in patients with vs without eDAC.

## Methods

### Study design

ENVISAGE-TAVI AF (NCT02943785) was a prospective, randomized, controlled, open-label, adjudicator-masked trial comparing edoxaban vs VKA in patients with AF after successful TAVR. The study design of the ENVISAGE-TAVI AF trial has been published in detail.^10^^,^^13^ The trial was conducted in accordance with the International Council for Harmonisation and the Declaration of Helsinki. The study was approved by the ethics committees and corresponding health authorities for all sites. All patients provided written informed consent before enrollment. An independent data and safety monitoring board reviewed all serious adverse events to ensure patients' safety.

### Study population

Patients aged ≥18 years with prevalent or incident AF after successful TAVR for severe aortic stenosis were eligible. Successful TAVR was defined as the implantation of any approved transcatheter bioprosthetic aortic valve into the proper anatomic location with the intended valve performance and without unresolved periprocedural complications. Patients were excluded if they were identified as having a high bleeding risk due to existing conditions (eg, active peptic ulcer with upper gastrointestinal bleeding within the 90 days before randomization, malignancy, recent brain or spinal surgery, or arteriovenous malformations). Patients were also excluded if they had an indication for dual antiplatelet therapy for >3 months. Additional inclusion and exclusion criteria have been previously published.^10^

### Study drug

Patients were randomized 1:1 to an antithrombotic regimen including edoxaban or VKA from April 2017 through January 2020. Edoxaban dose was reduced from 60 mg to 30 mg in patients meeting ≥1 of the eDAC per locally approved label (creatinine clearance [Cockcroft-Gault formula] 15 to ≤50 mL/min, body weight ≤60 kg [not part of the U.S. label], and use of P-glycoprotein inhibitors [not part of the U.S. label]). For VKA-treated patients, the target international normalized ratio was 2.0 to 3.0 (adjusted to 1.6-2.6 for patients aged ≥70 years in Japan). The prespecified use of an antiplatelet therapy in addition to edoxaban or VKAs was permitted and at the discretion of each treating physician in line with applicable guidelines.

### Endpoints

This post hoc analysis focused on net adverse clinical events (NACEs; composite all-cause death, myocardial infarction, ischemic stroke, systemic thromboembolic event, valve thrombosis, or MB), intracranial hemorrhage, ischemic stroke, all-cause death, cardiovascular (CV) death, non-CV death, MB, fatal MB, major nongastrointestinal bleeding, and MGIB. Net clinical outcome composite endpoints—defined as: 1) the composite of all-cause mortality, any stroke, and MB; 2) the composite of all-cause mortality, disabling stroke, and life-threatening bleeding; and 3) the composite of all-cause mortality, any stroke, and life-threatening bleeding—were also assessed.

### Statistical analysis

All analyses were performed on the safety analysis set during the on-treatment period, which included all randomized patients who received ≥1 dose of the study drug. The on-treatment period was defined as the duration of the treatment period and up to 3 days after the first interruption or discontinuation of study drug. Baseline demographic and clinical characteristics were presented using descriptive statistics and compared between patients with vs without eDAC at the time of randomization, as well as by the eDAC and age subgroup and by the eDAC and treatment group. Differences were tested using the unpaired Student’s *t*-test (continuous) or Fisher exact test (categorical) depending on the distribution of the variable. Annualized event rates were calculated as the number of patients experiencing a first event divided by the total person-time at risk while on treatment, expressed in years. The exposure time was defined as the period during which the patient was on treatment, plus 3 days following the last dose, and did not include any time during which the patient was not actively taking the drug.

Clinical outcomes were compared between patients with vs without eDAC and within age subgroups (<80 years vs ≥80 years). Age subgroups were selected based on a previous analysis of patients with AF aged ≥80 vs <80 years, which found that edoxaban 60 mg may have a greater impact on MGIB risk in patients ≥80 years of age.^11^ Edoxaban DAC were applied to patients on VKA who would have qualified for dose adjustment if they were on edoxaban. For each age subgroup, the effect of edoxaban vs VKA on clinical outcomes was assessed for patients with and without eDAC.

Cox proportional hazards regression models using the counting process approach were used to analyze time-to-event endpoints and to obtain the cause-specific HR with 2-sided 95% CIs for each variable. Each patient was included in the Cox regression analysis solely for the duration of their treatment period. When comparing oral anticoagulation groups, eDAC (yes vs no) and oral anticoagulant were included as the main effects with a multiplicative interaction between the 2 factors in the Cox model. The interaction *P* value and HRs were reported for each outcome with ≥5 events in both groups. The proportional hazards assumption of the Cox model was visually assessed using Schoenfeld residual plots. Fine and Gray regression models were used to assess the impact of the competing risk of death. All endpoints were adjusted for the competing risk of all-cause death, except CV death, which was adjusted for the competing risk of non-CV death, and non-CV death, which was adjusted for the competing risk of CV death. All-cause death and net clinical outcomes, including all-cause death in the composite endpoint, were not adjusted for any competing risks and corresponded to the results obtained from the Cox regression analysis. To assess the independent association of eDAC with outcomes, a supplemental analysis was performed with Cox regression models adjusted for baseline differences between eDAC groups including age, sex, history of hypertension, history of hypercholesterolemia, history of coronary artery bypass surgery, history of percutaneous coronary intervention within 30 days, history of gastrointestinal disorder, cigarette use, and platelet counts. Sensitivity analyses comparing eDAC (yes vs no) overall and within age subgroups were adjusted for baseline differences and accounted for the competing risk of death. Sensitivity analyses comparing treatment groups (edoxaban vs VKA) within age subgroups (≥80 and <80 years) accounted for the competing risk of death. These analyses were exploratory in nature with no adjustment prespecified for multiple comparisons. All statistical analyses were performed using SAS (version 9.4, SAS Institute).

## Results

### Patient characteristics

Of the 1,377 patients with AF post-TAVR, 637 (46%) met and 740 (54%) did not meet eDAC at randomization (Table 1). Among patients with eDAC, 540 (84.8%) had creatinine clearance ≤50 mL/min, 259 (40.7%) had a body weight of ≤60 kg, and 41 (6.4%) reported P-glycoprotein inhibitor use. Patients with vs without eDAC were older (83.8 ± 4.8 years vs 80.5 ± 5.5 years; *P* < 0.0001), had lower mean ± SD body weight (66.6 ± 16.6 kg vs 82.8 ± 14.9 kg; *P* < 0.0001), and more often had creatinine clearance ≤50 mL/min (81.3% vs 7.0%; *P* < 0.0001). CHA_2_DS_2_-VASc (congestive heart failure, hypertension, age ≥75 [doubled], diabetes, stroke [doubled], vascular disease, age 65 to 74, and sex category [female]) (4.7 ± 1.3 vs 4.3 ± 1.4; *P* < 0.0001) and Society of Thoracic Surgeons (STS) (6.0 ± 4.0 vs 3.9 ± 3.3; *P* < 0.0001) scores were higher in patients with vs without eDAC. Patients with vs without eDAC were more likely to have gastrointestinal disorder (41.1% vs 31.9%; *P* = 0.0004) and percutaneous coronary intervention performed ≤30 days before TAVR (6.1% vs 3.1%; *P* = 0.009).

**Table 1:** Baseline Demographic and Clinical Characteristics by eDAC (Safety Analysis Set)

	eDAC (n = 637)	No eDAC (n = 740)	*P* Value^a^
Age at enrollment, years, mean ± SD	83.8 ± 4.8	80.5 ± 5.5	**<0.0001**
<65	2 (0.3)	8 (1.1)	
≥65-<75	19 (3.0)	87 (11.8)	
≥75-<80	77 (12.1)	167 (22.6)	
≥80	539 (84.6)	478 (64.6)	
Sex			
Male	262 (41.1)	457 (61.8)	**<0.0001**
Female	375 (58.9)	283 (38.2)	
Race^b^			
White	465 (73.0)	681 (92.0)	**<0.0001**
Asian	146 (22.9)	33 (4.5)	**<0.0001**
Other	12 (1.9)	20 (2.7)	0.4
Weight, kg, mean ± SD	66.6 ± 16.6	82.8 ± 14.9	**<0.0001**
BMI, kg/m^2^, mean ± SD	25.6 ± 4.9	29.5 ± 5.4	**<0.0001**
CrCl, mL/min, mean ± SD	42.0 ± 14.1	72.3 ± 22.1	**<0.0001**
≤50 mL/min	518 (81.3)	52 (7.0)	**<0.0001**
Hemoglobin, g/L, mean ± SD	115.2 ± 94.1	116.1 ± 60.3	0.8
Platelet, 10^9^/L, mean ± SD	154.2 ± 53.4	162.3 ± 56.7	**0.007**
Ejection fraction, %, mean ± SD	55.2 ± 11.5	55.7 ± 11.3	0.5
HAS-BLED score, mean ± SD	1.6 ± 0.8	1.5 ± 0.8	0.05
CHA_2_DS_2_-VASc score, mean ± SD	4.7 ± 1.3	4.3 ± 1.4	**<0.0001**
STS score, mean ± SD	6.0 ± 4.0	3.9 ± 3.3	**<0.0001**
EuroScore I, mean ± SD	14.6 ± 10.3	11.4 ± 9.3	**<0.0001**
EuroScore II, mean ± SD	5.4 ± 4.5	3.9 ± 6.2	**<0.0001**
Type of AF^c^			
Paroxysmal AF	274 (43.0)	295 (39.9)	0.2
Persistent AF (>7 days but <1 year)	70 (11.0)	88 (11.9)	
Persistent AF (>1 year)	53 (8.3)	57 (7.7)	
Permanent AF	232 (36.4)	286 (38.6)	
Atrial flutter	6 (0.9)	12 (1.6)	
Medical history			
Stroke/TIA	112 (17.6)	121 (16.4)	0.6
Hypertension	571 (89.6)	687 (92.8)	**0.04**
Coronary artery disease	332 (52.1)	409 (55.3)	0.3
Hypercholesterolemia	423 (66.4)	541 (73.1)	**0.008**
Diabetes mellitus	219 (34.4)	287 (38.8)	0.09
Hospitalization for bleeding	25 (3.9)	35 (4.7)	0.5
Intracranial hemorrhage	10 (1.6)	7 (0.9)	0.3
Non-CNS systemic thromboembolic event	31 (4.9)	39 (5.3)	0.8
Peripheral artery disease	70 (11.0)	87 (11.8)	0.7
Carotid artery disease	38 (6.0)	58 (7.8)	0.2
COPD	84 (13.2)	115 (15.5)	0.2
MI	77 (12.1)	114 (15.4)	0.09
Prior major bleeding or predisposition to bleeding	61 (9.6)	58 (7.8)	0.3
CABG	42 (6.6)	82 (11.1)	**0.005**
PCI performed ≤30 d before TAVR	39 (6.1)	23 (3.1)	**0.009**
Gastrointestinal disorder	262 (41.1)	236 (31.9)	**0.0004**
APT before randomization	288 (45.2)	334 (45.1)	1.0
Previous PPI use	287 (45.1)	313 (42.3)	0.3
Pre-TAVR use			
VKA	268 (42.1)	365 (49.3)	**0.008**
DOAC	196 (30.8)	188 (25.4)	**0.03**
No pre-TAVR use of VKA or DOAC	173 (27.2)	187 (25.3)	0.5
Labile INR	49 (7.7)	59 (8.0)	0.9
Cigarette use (current or former)	169 (26.5)	276 (37.3)	**<0.0001**
Chronic drug usage	110 (17.3)	117 (15.8)	0.5
Excessive alcohol use	14 (2.2)	14 (1.9)	0.7
Edoxaban group	319 (50.1)	374 (50.5)	0.9
Edoxaban initial dose^d^			
30 mg	309 (96.9)	11 (2.9)	**<0.0001**
60 mg	9 (2.8)	361 (96.5)	**<0.0001**
eDAC at randomization^e^			
CrCl ≤50 mL/min	540 (84.8)	0	NA
Weight ≤60 kg	259 (40.7)	0	NA
P-glycoprotein inhibitor use	41 (6.4)	0	NA
Number of eDAC at randomization			
1	449 (70.5)	0	NA
2	173 (27.2)	0	NA
3	15 (2.4)	0	NA

Values are n (%) unless otherwise noted. Categorical variables were presented as frequencies and percentages, and continuous variables were presented as means ± SD. Percentage calculations were based on the total number of patients in the analysis. **Bolded***P* values are significant (*P* < 0.05).

AF = atrial fibrillation; APT = antiplatelet therapy; BMI = body mass index; CABG = coronary artery bypass graft; CHA_2_DS_2_-VASc, Congestive heart failure, Hypertension, Age ≥75 (doubled), Diabetes, Stroke (doubled), Vascular disease, Age 65 to 74, and Sex category (female); CNS = central nervous system; COPD = chronic obstructive pulmonary disease; CrCl = creatinine clearance; DOAC = direct oral anticoagulant; eDAC = edoxaban dose adjustment criteria; HAS-BLED = Hypertension, Abnormal renal and liver function, Stroke, Bleeding, Labile INR, Elderly, Drugs or alcohol; INR = international normalized ratio; MI = myocardial infarction; NA = not applicable; PCI = percutaneous coronary intervention; PPI = proton pump inhibitor; STS = Society of Thoracic Surgeons; TAVR = transcatheter aortic valve replacement; TIA = transient ischemic attack; VKA = vitamin K antagonist.

a Differences were tested using the unpaired Student’s *t*-test or Fisher exact test depending on the distribution of the variable.

b Race was analyzed as specified on the case report form including ‘Other’ representing those that selected ‘Other’ as a category. Smaller categories with fewer than 10 patients (Black or African American [n = 5], American Indian/Alaska Native [n = 1]) and patients for whom race was not recorded (n = 14) were not reported by eDAC subgroups.

c *P* value for AF type is paroxysmal vs nonparoxysmal.

d Edoxaban initial dose was 0 mg in 2 patients from the no eDAC group where a >3-day treatment interruption was recorded and 0 mg in 1 patient from the eDAC group where a >3-day treatment interruption was recorded.

e Twenty patients were not treated in line with eDAC per locally approved label as defined for randomization (9 with eDAC at randomization received 60 mg as the first dose and 11 without eDAC at randomization received 30 mg as the first dose). This might be driven by changed eDAC by the time of first dose.

Baseline demographics and clinical characteristics were similar between treatment arms (edoxaban: n = 693; VKA, n = 684) for patients with and without eDAC (Supplemental Table 1). Patients with vs without eDAC who were on VKA had significantly lower mean ± SD percent time in therapeutic range for the international normalized ratio (61.4 ± 24.0 vs 65.3 ± 21.6; *P* = 0.04). Antiplatelet therapy during the study was well balanced among patients with vs without eDAC (54.5% vs 58.8%), age subgroups, and treatment arms (Supplemental Table 2). The mean ± SD on-treatment follow-up duration was 1.3 ± 0.8 years.

Among patients aged <80 years (n = 360), 98 (27%) met and 262 (73%) did not meet eDAC (Table 2). Those with vs without eDAC had a lower mean ± SD body weight (73.9 ± 22.5 kg vs 88.4 ± 17.6 kg; *P* < 0.0001) and more often had creatinine clearance ≤50 mL/min (65.3% vs 2.7%; *P* < 0.0001). In addition, patients with vs without eDAC were more often female (51.0% vs 37.8%; *P* = 0.03), had a higher mean CHA_2_DS_2_-VASc score (4.7 ± 1.4 vs 4.2 ± 1.4; *P* = 0.007), and a higher mean STS score (5.2 ± 3.7 vs 3.5 ± 3.0; *P* < 0.0001). The median follow-up times for patients aged <80 years with and without eDAC were 1.3 and 1.5 years, respectively.

**Table 2:** Demographic and Baseline Characteristics by eDAC Within Age Subgroups (Safety Analysis Set)

	Age <80 y		*P* Value	Age ≥80 y		*P* Value^a^
	eDAC (n = 98)	No eDAC (n = 262)		eDAC (n = 539)	No eDAC (n = 478)	
Age at enrollment, y, mean ± SD	75.9 ± 3.7	74.8 ± 4.1	**0.02**	85.2 ± 3.4	83.7 ± 3.0	**<0.0001**
<65	2 (2.0)	8 (3.1)		0	0	
≥65 to <75	19 (19.4)	87 (33.2)		0	0	
≥75 to <80	77 (78.6)	167 (63.7)		0	0	
≥80	0	0		539 (100)	478 (100)	
Sex						
Male	48 (49.0)	163 (62.2)	**0.03**	214 (39.7)	294 (61.5)	**<0.0001**
Female	50 (51.0)	99 (37.8)		325 (60.3)	184 (38.5)	
Race						
White	71 (72.4)	245 (93.5)	**<0.0001**	394 (73.1)	436 (91.2)	**<0.0001**
Asian	19 (19.4)	7 (2.7)	**<0.0001**	127 (23.6)	26 (5.4)	**<0.0001**
Other	3 (3.1)	7 (2.7)	1.0	9 (1.7)	13 (2.7)	0.3
Weight, kg, mean ± SD	73.9 ± 22.5	88.4 ± 17.6	**<0.0001**	65.3 ± 15.0	79.7 ± 12.1	**<0.0001**
BMI, kg/m^2^, mean ± SD	27.5 ± 6.4	31.1 ± 6.5	**<0.0001**	25.2 ± 4.5	28.6 ± 4.4	**<0.0001**
CrCl, mL/min, mean ± SD	50.2 ± 19.6	83.4 ± 26.6	**<0.0001**	40.5 ± 12.3	66.2 ± 16.3	**<0.0001**
≤50 mL/min	64 (65.3)	7 (2.7)	**<0.0001**	454 (84.2)	45 (9.4)	**<0.0001**
Hemoglobin, g/L, mean ± SD	120.5 ± 125.4	117.5 ± 57.2	0.8	114.2 ± 87.4	115.3 ± 62.1	0.8
Platelet, 10^9^/L, mean ± SD	169.9 ± 57.3	168.4 ± 56.5	0.8	151.4 ± 52.2	159.0 ± 56.5	**0.03**
Ejection fraction, %, mean ± SD	51.5 ± 12.7	54.2 ± 11.4	0.06	55.9 ± 11.2	56.5 ± 11.2	0.4
HAS-BLED score, mean ± SD	1.7 ± 0.8	1.5 ± 0.8	0.08	1.6 ± 0.8	1.6 ± 0.7	0.3
CHA_2_DS_2_-VASc score, mean ± SD	4.7 ± 1.4	4.2 ± 1.4	**0.007**	4.7 ± 1.3	4.4 ± 1.3	**0.0007**
STS score, mean ± SD	5.2 ± 3.7	3.5 ± 3.0	**<0.0001**	6.1 ± 4.1	4.1 ± 3.5	**<0.0001**
EuroScore I, mean ± SD	11.5 ± 9.1	9.5 ± 8.3	0.06	15.2 ± 10.4	12.5 ± 9.6	**<0.0001**
EuroScore II, mean ± SD	4.7 ± 4.6	4.4 ± 9.3	0.7	5.5 ± 4.5	3.6 ± 3.4	**<0.0001**
Type of AF^b^						
Paroxysmal AF	47 (48.0)	118 (45.0)	0.6	227 (42.1)	177 (37.0)	0.1
Persistent AF (>7 days but <1 y)	11 (11.2)	32 (12.2)		59 (10.9)	56 (11.7)	
Persistent AF (>1 y)	4 (4.1)	19 (7.3)		49 (9.1)	38 (7.9)	
Permanent AF	34 (34.7)	88 (33.6)		198 (36.7)	198 (41.4)	
Atrial flutter	2 (2.0)	5 (1.9)		4 (0.7)	7 (1.5)	
Medical History						
Stroke/TIA	18 (18.4)	38 (14.5)	0.4	94 (17.4)	83 (17.4)	1.0
Hypertension	85 (86.7)	240 (91.6)	0.2	486 (90.2)	447 (93.5)	0.07
Coronary artery disease	52 (53.1)	144 (55.0)	0.8	280 (51.9)	265 (55.4)	0.3
Hypercholesterolemia	69 (70.4)	197 (75.2)	0.4	354 (65.7)	344 (72.0)	**0.04**
Diabetes mellitus	47 (48.0)	133 (50.8)	0.7	172 (31.9)	154 (32.2)	0.9
Hospitalization for bleeding	6 (6.1)	7 (2.7)	0.1	19 (3.5)	28 (5.9)	0.1
Intracranial hemorrhage	2 (2.0)	2 (0.8)	0.3	8 (1.5)	5 (1.0)	0.6
Non-CNS systemic thromboembolic event	6 (6.1)	14 (5.3)	0.8	25 (4.6)	25 (5.2)	0.7
Peripheral artery disease	8 (8.2)	32 (12.2)	0.3	62 (11.5)	55 (11.5)	1.0
Carotid artery disease	5 (5.1)	22 (8.4)	0.4	33 (6.1)	36 (7.5)	0.4
COPD	21 (21.4)	53 (20.2)	0.9	63 (11.7)	62 (13.0)	0.6
MI	17 (17.3)	52 (19.8)	0.7	60 (11.1)	62 (13.0)	0.4
Prior major bleeding or predisposition to bleeding	9 (9.2)	18 (6.9)	0.5	52 (9.6)	40 (8.4)	0.5
CABG	11 (11.2)	50 (19.1)	0.08	31 (5.8)	32 (6.7)	0.6
PCI performed ≤30 days before TAVR	5 (5.1)	8 (3.1)	0.4	34 (6.3)	15 (3.1)	**0.02**
Gastrointestinal disorder	39 (39.8)	82 (31.3)	0.1	223 (41.4)	154 (32.2)	**0.003**
APT before randomization	47 (48.0)	121 (46.2)	0.8	241 (44.7)	213 (44.6)	1.0
Previous PPI use	45 (45.9)	116 (44.3)	0.8	242 (44.9)	197 (41.2)	0.3
Pre-TAVR use						
VKA	36 (36.7)	138 (52.7)	**0.009**	232 (43.0)	227 (47.5)	0.2
DOAC	33 (33.7)	66 (25.2)	0.1	163 (30.2)	122 (25.5)	0.1
No pre-TAVR use of VKA or DOAC	29 (29.6)	58 (22.1)	0.2	144 (26.7)	129 (27.0)	0.9
Labile INR	10 (10.2)	20 (7.6)	0.5	39 (7.2)	39 (8.2)	0.6
Cigarette use (current or former)	38 (38.8)	108 (41.2)	0.7	131 (24.3)	168 (35.1)	**0.0002**
Chronic drug usage	14 (14.3)	37 (14.1)	1.0	96 (17.8)	80 (16.7)	0.7
Excessive alcohol use	3 (3.1)	8 (3.1)	1.0	11 (2.0)	6 (1.3)	0.5
Edoxaban group	52 (53.1)	132 (50.4)	0.7	267 (49.5)	242 (50.6)	0.8
Edoxaban initial dose						
30 mg	49 (94.2)	4 (3.0)	**<0.0001**	260 (97.4)	7 (2.9)	**<0.0001**
60 mg	3 (5.8)	127 (96.2)	**<0.0001**	6 (2.2)	234 (96.7)	**<0.0001**

Values are n (%) unless otherwise noted. Categorical variables were presented as frequencies and percentages, and continuous variables were presented as means ± SD. Percentage calculations were based on the total number of patients in the analysis.

Abbreviations as in Table 1.

a Differences were tested using the unpaired Student’s *t*-test or Fisher exact test depending on the distribution of the variable.

b *P* value for AF type is paroxysmal vs nonparoxysmal.

Among patients aged ≥80 years (n = 1,017), 539 (53%) met and 478 (47%) did not meet ≥1 eDAC (Table 2). Those with vs without eDAC had a lower mean ± SD body weight (65.3 ± 15.0 kg vs 79.7 ± 12.1 kg; *P* < 0.0001) and more often had creatinine clearance ≤50 mL/min (84.2% vs 9.4%; *P* < 0.0001). Patients with vs without eDAC were more often female (60.3% vs 38.5%; *P* < 0.0001), had a higher mean CHA_2_DS_2_-VASc (4.7 ± 1.3 vs 4.4 ± 1.3; *P* = 0.0007), and STS scores (6.1 ± 4.1 vs 4.1 ± 3.5; *P* < 0.0001). Among patients aged ≥80 years, the median follow-up time was 1.2 years for those with or without eDAC.

### Clinical outcomes

**Figure 1:**
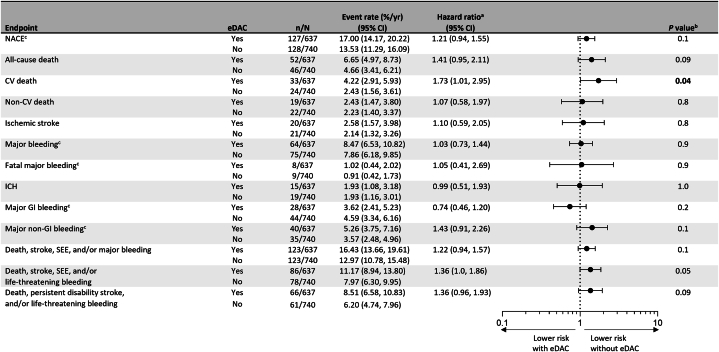
**Annualized On-Treatment Event Rates and Cox Regression of Endpoints Comparing Patients With and Without Edoxaban Dose Adjustment Criteria** ^a^HRs were only calculated for outcomes with ≥5 events in both groups. ^b^Significant *P* values were bolded. ^c^The ISTH definition was used. CV = cardiovascular; eDAC = edoxaban dose adjustment criteria; GI = gastrointestinal; ICH = intracranial hemorrhage; ISTH = International Society on Thrombosis and Haemostasis; NACE = net adverse clinical events; SEE = systemic embolic events.

Patients with vs without eDAC had significantly higher rates of CV death (HR: 1.73; 95% CI: 1.01-2.95; *P* = 0.045) (Figure 1, Supplemental Table 3). There were no significant differences between patients with vs without eDAC for the remaining outcomes, including the rates of NACE (HR: 1.21; 95% CI: 0.94-1.55; *P* = 0.14), all-cause death (HR: 1.41; 95% CI: 0.95-2.11; *P* = 0.09), MB (HR: 1.03; 95% CI: 0.73-1.44; *P* = 0.9), fatal MB (HR: 1.05; 95% CI: 0.41-2.69; *P* = 0.9), or MGIB (HR: 0.74; 95% CI: 0.46-1.20; *P* = 0.2) (Figure 1). The risk of major nongastrointestinal bleeding was numerically higher in patients with vs without eDAC (HR: 1.43; 95% CI: 0.91-2.26; *P* = 0.1). There were no significant differences in the rates of net clinical outcome including death, ischemic stroke, systemic embolic events, and/or MB (HR: 1.22; 95% CI: 0.94-1.57; *P* = 0.1), death, stroke, systemic embolic events, and/or life-threatening bleeding (HR: 1.36; 95% CI: 1.00-1.86; *P* = 0.05), or death, persistent disability stroke, and/or life-threatening bleeding (HR: 1.36; 95% CI: 0.96-1.93; *P* = 0.09).

**Figure 2:**
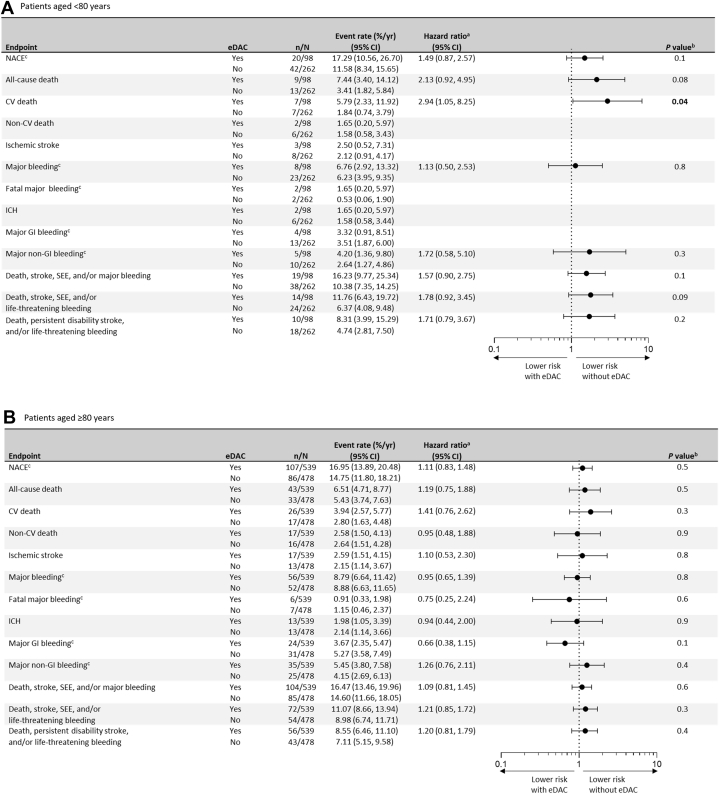
**Annualized On-Treatment Event Rates and Cox Regression of Endpoints Comparing Patients With vs Without Edoxaban Dose Adjustment Criteria** (A) Patients aged <80 years and (B) patients aged ≥80 years. ^a^HRs were only calculated for outcomes with ≥5 events in both groups. ^b^Significant *P* values were bolded. ^c^The ISTH definition was used. Abbreviations as in Figure 1.

Among patients aged <80 years, the risk of CV death was higher in those with vs without eDAC (HR: 2.94; 95% CI: 1.05-8.25; *P* = 0.04) (Figure 2A). Similarly, in patients aged ≥80 years with vs without eDAC, a numerically higher risk of cardiovascular death was seen (HR: 1.41; 95% CI: 0.76-2.62; *P* = 0.3) (Figure 2B). In line with the overall results, there were no significant differences within each age subgroup for the remaining outcomes for patients with vs without eDAC. In a sensitivity analysis assessing the independent association of eDAC with outcomes and the impact of the competing risk of death, estimates of the risk were consistent with the unadjusted analysis (Supplemental Figures 1 and 2).

### Clinical outcomes by treatment arm

**Figure 3:**
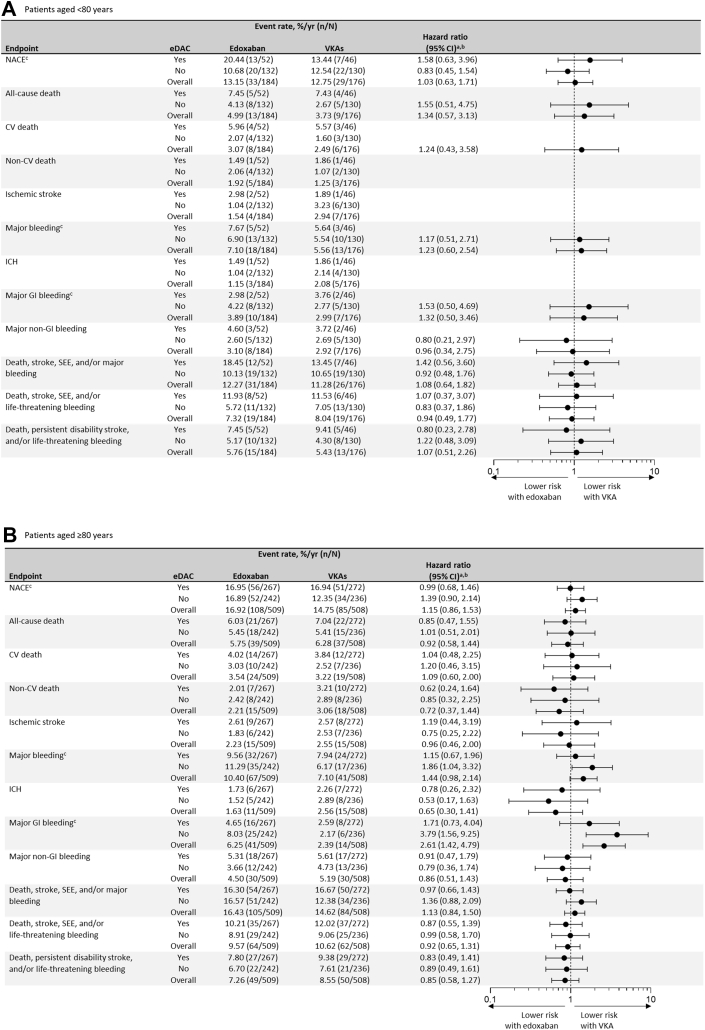
**Clinical Outcomes in Patients Receiving Edoxaban vs Vitamin K Antagonists Stratified by the Presence or Absence of Edoxaban Dose Adjustment Criteria** (A) Aged <80 years and (B) aged ≥80 years. ^a^HRs were only calculated for outcomes with ≥5 events in both groups. ^b^There were no statistically significant interactions assessing for a differential treatment effect comparing edoxaban vs VKA by eDAC (all *P* ≥ 0.20). ^c^The ISTH definition was used. VKA = vitamin K antagonist; other abbreviations as in Figure 1.

Among patients aged <80 years and ≥80 years, there were no statistically significant interactions between the presence or absence of eDAC and treatment with edoxaban vs VKAs for the outcomes assessed (Figures 3A and 3B). In patients aged <80 years, there were no significant treatment differences comparing edoxaban vs VKA (Figure 3A). However, in patients aged ≥80 years, treatment with edoxaban vs VKA was associated with numerically higher annualized event rates and risk of MB (HR: 1.44; 95% CI: 0.98-2.14) and statistically significantly higher risk of MGIB (HR: 2.61; 95% CI: 1.42-4.79) (Figure 3B), irrespective of eDAC. These event rates and risks comparing edoxaban vs VKA treatment were the highest among patients without eDAC (MB, HR: 1.86; 95% CI: 1.04-3.32; MGIB, HR: 3.79; 95% CI: 1.56-9.25). When evaluating edoxaban-treated patients, the rate of MGIB almost doubled among those aged ≥80 years without vs with eDAC (8.03%/year vs 4.65%/year). This effect was also seen in patients aged <80 years without vs with eDAC (4.22%/year vs 2.98%/year). Conversely, when evaluating patients treated with VKA, similar MGIB event rates were observed between patients without vs with eDAC, irrespective of age group (<80 years: 2.77%/year vs 3.76%/year; ≥80 years: 2.17%/year vs 2.59%/year). The rate of major nongastrointestinal bleeding in edoxaban vs VKA-treated patients was numerically lower (HR: 0.86; 95% CI: 0.51-1.43). This effect was consistent among those with (HR: 0.91; 95% CI: 0.47-1.79) and without eDAC (HR: 0.79; 95% CI: 0.36-1.74). In a sensitivity analysis assessing the impact of the competing risk of death on endpoints that do not include all-cause death, estimates of the risk were consistent with the primary analysis (Supplemental Figure 3).

## Discussion

The main findings of this post hoc on-treatment analysis of the ENVISAGE-TAVI AF trial on the clinical implications of the presence of eDAC and age can be summarized as follows: 1) among patients aged <80 years, those with vs without eDAC had higher CV mortality and 2) treatment with edoxaban vs VKAs was associated with numerically higher rates of MB and MGIB in patients aged ≥80 years without eDAC (Central Illustration). These results suggest that patients aged <80 years with AF post-TAVR may be a more vulnerable population at a higher risk of CV death. In addition, adjusting edoxaban dosing from 60 mg to 30 mg in patients aged ≥80 years with AF after successful TAVR (regardless of eDAC) may reduce the likelihood of MB and MGIB.

**Central Illustration:**
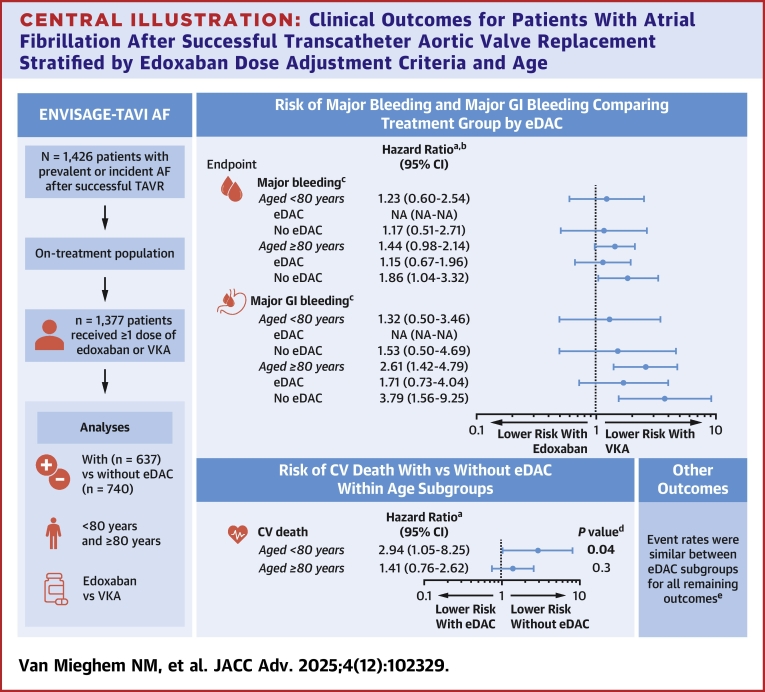
**Clinical Outcomes for Patients With Atrial Fibrillation After Successful Transcatheter Aortic Valve Replacement Stratified by Edoxaban Dose Adjustment Criteria and Age** This post hoc, on-treatment analysis of the ENVISAGE-TAVI AF trial assessed whether the presence or absence of eDAC and age (<80 vs ≥ 80 years) affected clinical outcomes. Edoxaban dose was to be reduced from 60 mg to 30 mg in patients with AF who met ≥1 criteria: CrCl 15 to ≤50 mL/min; body weight ≤60 kg (not part of the U.S. label); concomitant P-glycoprotein inhibitors per local label (not part of the U.S. label). Edoxaban DAC also applied to patients on VKA. ^a^HRs were only calculated for outcomes with ≥5 events in both groups. ^b^There were no statistically significant interactions assessing for a differential treatment effect comparing edoxaban vs VKA by eDAC (all *P* ≥ 0.20). ^c^ISTH definition was used. ^d^Significant *P* values were bolded. ^e^Data not shown. AF = atrial fibrillation; CrCl = creatinine clearance; CV = cardiovascular; eDAC = edoxaban dose adjustment criteria; GI = gastrointestinal; HR = hazard ratio; ISTH = International Society on Thrombosis and Haemostasis; NA = not applicable; TAVR = transcatheter aortic valve replacement; VKA = vitamin K antagonist.

Specifically, this analysis found that most deaths among patients with and without eDAC were due to CV causes, which was primarily driven by patients aged <80 years. Among patients aged <80 years, those with eDAC had higher mean CHA_2_DS_2_-VASc score, and lower mean body weight and creatinine clearance compared with patients without eDAC. Previous studies demonstrated that both comorbidities and renal impairment in patients with AF may lead to a significantly higher risk of CV death.^14^^,^^15^ Furthermore, in a mortality analysis of ENGAGE AF-TIMI 48, most deaths were CV-related in patients with nonvalvular AF treated with edoxaban vs warfarin, consistent with the results from this post hoc analysis.^16^ Previous research has shown that patients with AF who were frail had a higher risk of CV death than those who were not.^17^ Although this prior analysis did not assess frailty, patients aged <80 years with eDAC represented a more vulnerable phenotype at risk of CV death due to an accumulation of deficits. Even though the ENGAGE AF-TIMI 48 study previously suggested that edoxaban 30 mg may provide similar effectiveness in reducing ischemic events as VKA,^11^ an individualized approach may be warranted when determining the appropriate DOAC strategy in patients <80 years with eDAC.

Rates of NACE, CV death, and all-cause death were similar among patients aged ≥80 years on edoxaban vs VKA without eDAC; however, there were numerically higher rates of MB and MGIB. Notably, the MGIB rates almost doubled in patients taking edoxaban without vs with eDAC (8.03%/year vs 4.65%/year). Conversely, despite observing higher rates of MB and MGIB, the rates of major nongastrointestinal bleeding were numerically lower with edoxaban vs VKA. Furthermore, this particular subset of patients had significantly higher rates of gastrointestinal disorders (41.4% vs 32.2%; *P* = 0.003). Consistent with previous findings, these results may suggest that gastrointestinal disorders contribute to bleeding risk in patients aged ≥80 years of age without eDAC.^18^ Moreover, older patients on anticoagulants may be more susceptible to bleeding complications due to age-related changes in the gastrointestinal tract.^19^ The FRAIL-AF trial similarly showed higher rates of bleeding in patients aged ≥75 years after switching from well-managed VKA to a DOAC vs continuing on VKA.^5^ Therefore, physicians should focus on patients with AF after TAVR aged ≥80 years who have a susceptibility to and a higher risk of MB and MGIB by optimizing anticoagulant dose.

This higher rate of bleeding may also be explained by an association between age and bleeding risk in older patients without eDAC. In a previous analysis of bleeding risk within age subgroups from the ENGAGE AF-TIMI 48 trial, patients aged ≥75 years had fewer MB events on edoxaban 30 mg vs warfarin, regardless of eDAC.^11^ Event rates were similar between treatment groups for patients aged ≥75 years without eDAC receiving edoxaban 60 mg vs warfarin.^11^ Post hoc analyses of ENGAGE AF-TIMI 48 data demonstrated that increasing edoxaban concentration resulted in a steep increase of MB risk and a gradual decrease in the risk of ischemic stroke or systemic embolic event, and that patients with AF aged ≥80 years achieved higher edoxaban concentrations and a more pronounced anticoagulant effect compared with younger patients who received the same dose.^20^^,^^21^ Our analysis concurs with these findings and suggests that the higher risk of bleeding events in older patients treated with edoxaban vs VKA may be mitigated by the 30-mg edoxaban dose without an impact on efficacy.

Finally, this analysis shows that the efficacy of edoxaban 30 mg was maintained among patients aged ≥80 years with AF after TAVR as the risk of ischemic stroke was similar when comparing edoxaban vs VKA in patients with eDAC. This was consistent with previous findings from ENGAGE AF-TIMI 48 where rates of ischemic stroke or systemic embolic event were similar between edoxaban vs VKA among patients aged ≥80 years with and without eDAC.^21^ Furthermore, the net clinical outcome was 22% lower among older patients on edoxaban vs VKA, regardless of eDAC.^21^ These findings collectively support adjusting edoxaban dose to 30 mg for patients aged ≥80 years with AF after TAVR to minimize bleeding risk without losing efficacy against ischemic stroke.

### Study Limitations

There are several limitations to this post hoc analysis of the ENVISAGE-TAVI AF trial. This analysis consisted of a primarily elderly population (mean age: 82 ± 5.4 years) and patients aged <80 years were under-represented. The treatment discontinuation rate during the primary ENVISAGE-TAVI AF trial was 30% in patients taking edoxaban and 41% in those on VKA; however, the mean duration of follow-up during the full study period in patients on edoxaban and VKA was 1.5 years, respectively.^10^ Discontinuation rates >30% have similarly been reported in other randomized controlled trials comparing DOAC with VKA (ie, GALILEO: 37%; ENGAGE AF-TIMI 48: 33%).^22^^,^^23^ It should also be noted that the very low rates of some events (eg, ischemic stroke) limited our ability to analyze differences between groups. This was an open-label study, which may have introduced reporting biases regarding trial outcomes. In addition, this was a post hoc analysis and was not powered to assess the effect of edoxaban vs VKA in patients with and without eDAC. Therefore, the results should be considered exploratory. Finally, edoxaban plasma concentrations and antifactor Xa activity were not measured, limiting the ability to directly assess the impact of age on edoxaban levels in patients with and without eDAC. Other key limitations of the ENVISAGE-TAVI AF trial are published and should be considered when interpreting the results of the current analysis.^10^

## Conclusions

In this post hoc analysis of ENVISAGE-TAVI AF, treatment with edoxaban vs VKAs was associated with numerically higher rates of MB and MGIB, particularly among patients aged ≥80 years who did not meet eDAC. Therefore, adjusting edoxaban dosing from 60 mg to 30 mg in patients aged ≥80 years with AF after TAVR, regardless of eDAC, may reduce the likelihood of MB and MGIB without increasing the risk of thromboembolic events.

Perspectives **COMPETENCY IN MEDICAL KNOWLEDGE:** The presence of eDAC identify a more vulnerable population. Patients aged ≥80 years with AF after TAVR taking the full dose of edoxaban 60 mg for ischemic stroke prevention have a higher risk for MB and MGIB compared with VKAs. Adjusting edoxaban dosing from 60 mg to 30 mg in patients aged ≥80 years, regardless of meeting eDAC, may reduce the likelihood of MB and MGIB.

**TRANSLATIONAL OUTLOOK:** In this post hoc analysis of the ENVISAGE-TAVI AF trial, treatment with edoxaban vs VKAs was associated with numerically higher rates of MB and MGIB. However, most patients in this analysis were elderly (≥80 years of age). Studies including octogenarians with nonvalvular AF have already suggested lower bleeding rates without increased thromboembolic risk with edoxaban 30 mg. Age (≥80 years) may be a clinically relevant reason for edoxaban dose adjustment.

## Funding support and author disclosures

This study was funded by 10.13039/501100002336Daiichi Sankyo. Dr Van Mieghem reports grants or contracts from Abbott Vascular, Abiomed, Boston Scientific, Daiichi Sankyo, Edwards Lifesciences, Medtronic, PulseCath BV, and Siemens. Dr Hengstenberg is a clinical proctor for Boston Scientific and Edwards Lifesciences and reports payment for speaker bureaus, support for attending meetings, and advisory board participation from Daiichi Sankyo as well as institutional funding from 10.13039/100011949Abbott Vascular, 10.13039/100002429Amgen, 10.13039/501100008877Biosensors, 10.13039/501100005035Biotronik, 10.13039/100001003Boehringer Ingelheim, Boston Scientific, 10.13039/501100002336Daiichi Sankyo, 10.13039/100006520Edwards Lifesciences, Ferrer, 10.13039/100004374Medtronic, 10.13039/100004336Novartis, 10.13039/100004320Philips, 10.13039/100004340Siemens, and 10.13039/501100008645Terumo. Dr Chen, Dr Van Zyl, Mr Kimura, and Dr Unverdorben are employees of Daiichi Sankyo. Dr Mehran reports research grants to institution and support for attending meetings from Bayer and Daiichi Sankyo, and consulting fees from Daiichi Sankyo. Dr Nicolas is supported by a grant from the 10.13039/100000002National Institute of Health/10.13039/100000050National Heart, Lung, and Blood Institute (R38). Dr Dangas reports research grants to institution and support for attending meetings from Bayer and Daiichi Sankyo, and consulting fees from Daiichi Sankyo. All other authors have reported that they have no relationships relevant to the contents of this paper to disclose.
