# Supplementary material for: Edoxaban Dose Adjustment and Age-Associated Outcomes in Patients With Atrial Fibrillation Post-Transcatheter Aortic Valve Replacement
Source: JACC Adv. 2025 Nov 19;4(12):102329. doi: 10.1016/j.jacadv.2025.102329 (PMC12805168; doi:10.1016/j.jacadv.2025.102329)
Supplement: Supplemental Tables 1 to 3 and Supplementary figures 1 to — 3 [file mmc1.pdf]

SUPPLEMENTAL TABLE 1. Baseline Demographic and Clinical Characteristics by Anticoagulant Use (Safety Analysis Set)

Parameter	eDAC[a] Edoxaban (n = 319)	eDAC[a] VKA (n = 318)	*P* value	No eDAC[a] Edoxaban (n = 374)	No eDAC[a] VKA (n = 366)	*P* value[b]
**Age at enrollment, years, mean ± SD**	83.8 ± 4.9	83.8 ± 4.8	0.8	80.5 ± 5.3	80.6 ± 5.6	0.9
<65	1 (0.3)	1 (0.3)		4 (1.1)	4 (1.1)	
≥65 to <75	11 (3.4)	8 (2.5)		42 (11.2)	45 (12.3)	
≥75 to <80	40 (12.5)	37 (11.6)		86 (23.0)	81 (22.1)	
≥80	267 (83.7)	272 (85.5)		242 (64.7)	236 (64.5)	
**Sex**						
Male	130 (40.8)	132 (41.5)	0.9	225 (60.2)	232 (63.4)	0.4
Female	189 (59.2)	186 (58.5)		149 (39.8)	134 (36.6)	
**Race**						
White	234 (73.4)	231 (72.6)	0.9	343 (91.7)	338 (92.3)	0.8
Asian	73 (22.9)	73 (23.0)	1.0	17 (4.5)	16 (4.4)	1.0
Other	4 (1.3)	8 (2.5)	0.3	9 (2.4)	11 (3.0)	0.7
**Weight, kg, mean ± SD**	65.3 ± 15.7	67.9 ± 17.4	**0.049**	82.5 ± 15.8	83.0 ± 13.9	0.7
**BMI, kg/m^2, mean ± SD**	25.3 ± 4.9	25.9 ± 4.9	0.1	29.4 ± 5.6	29.6 ± 5.1	0.6
**CrCl, mL/min, mean ± SD**	41.2 ± 12.9	42.7 ± 15.2	0.2	72.1 ± 22.1	72.5 ± 22.1	0.8
≤50 mL/min	267 (83.7)	251 (78.9)	0.1	33 (8.8)	19 (5.2)	0.06
**Hemoglobin, g/L, mean ± SD**	113.2 ± 70.2	117.1 ± 113.2	0.6	115.6 ± 49.2	116.6 ± 70.0	0.8
**Platelet, 10^9/L, mean ± SD**	154.8 ± 54.6	153.7 ± 52.2	0.8	161.7 ± 55.5	163.0 ± 57.8	0.8
**Ejection fraction, %, mean ± SD**	55.1 ± 11.4	55.3 ± 11.6	0.8	55.5 ± 11.8	55.9 ± 10.9	0.7

**Labile INR**	25 (7.8)	24 (7.5)	1.0	26 (7.0)	33 (9.0)	0.3
**HAS-BLED score, mean ± SD**	1.7 ± 0.8	1.6 ± 0.8	0.2	1.6 ± 0.8	1.5 ± 0.7	0.5
**CHA_2DS_2-VASc score, mean ± SD**	4.7 ± 1.4	4.6 ± 1.2	0.5	4.3 ± 1.3	4.3 ± 1.4	0.6
**STS score, mean ± SD**	5.9 ± 3.7	6.1 ± 4.4	0.5	3.9 ± 3.1	4.0 ± 3.5	0.7
**EuroScore, mean ± SD**						
I	14.7 ± 10.5	14.5 ± 10.1	0.8	11.3 ± 8.8	11.6 ± 9.7	0.6
II	5.3 ± 4.1	5.5 ± 4.9	0.5	3.8 ± 6.1	4.0 ± 6.3	0.6
**Type of AF[c]**						
Paroxysmal AF	144 (45.1)	130 (40.9)	0.3	137 (36.6)	158 (43.2)	0.07
Persistent AF (>7 days but <1 year)	37 (11.6)	33 (10.4)		42 (11.2)	46 (12.6)	
Persistent AF (>1 year)	24 (7.5)	29 (9.1)		32 (8.6)	25 (6.8)	
Permanent AF	111 (34.8)	121 (38.1)		159 (42.5)	127 (34.7)	
Atrial flutter	1 (0.3)	5 (1.6)		4 (1.1)	8 (2.2)	
**Medical History**						
Stroke/TIA	66 (20.7)	46 (14.5)	**0.048**	54 (14.4)	67 (18.3)	0.2
Hypertension	279 (87.5)	292 (91.8)	0.09	350 (93.6)	337 (92.1)	0.5
Coronary artery disease	173 (54.2)	159 (50.0)	0.3	198 (52.9)	211 (57.7)	0.2
Hypercholesterolemia	207 (64.9)	216 (67.9)	0.5	266 (71.1)	275 (75.1)	0.2
Diabetes mellitus	107 (33.5)	112 (35.2)	0.7	153 (40.9)	134 (36.6)	0.3
Hospitalization for bleeding	14 (4.4)	11 (3.5)	0.7	20 (5.3)	15 (4.1)	0.5
Non-CNS systemic thromboembolic event	19 (6.0)	12 (3.8)	0.3	14 (3.7)	25 (6.8)	0.07
Peripheral artery disease	28 (8.8)	42 (13.2)	0.08	46 (12.3)	41 (11.2)	0.7
Carotid artery disease	16 (5.0)	22 (6.9)	0.3	32 (8.6)	26 (7.1)	0.5

COPD	44 (13.8)	40 (12.6)	0.7	58 (15.5)	57 (15.6)	1.0
MI	42 (13.2)	35 (11.0)	0.5	54 (14.4)	60 (16.4)	0.5
Prior major bleeding or predisposition to bleeding	32 (10.0)	29 (9.1)	0. 8	32 (8.6)	26 (7.1)	0.5
Intracranial hemorrhage	5 (1.6)	5 (1.6)	1.0	6 (1.6)	1 (0.3)	0.1
CABG	23 (7.2)	19 (6.0)	0.6	44 (11.8)	38 (10.4)	0.6
PCI performed ≤30 days before TAVR	21 (6.6)	18 (5.7)	0.7	13 (3.5)	10 (2.7)	0.7
Gastrointestinal disorder	137 (42.9)	125 (39.3)	0.4	123 (32.9)	113 (30.9)	0.6
**APT prior to randomization**	144 (45.1)	144 (45.3)	1.0	165 (44.1)	169 (46.2)	0.6
**Previous PPI use**	156 (48.9)	131 (41.2)	0.06	146 (39.0)	167 (45.6)	0.07
**Pre-TAVR use**						
VKA	136 (42.6)	132 (41.5)	0.8	169 (45.2)	196 (53.6)	**0.03**
DOAC	94 (29.5)	102 (32.1)	0.5	104 (27.8)	84 (23.0)	0.2
**No pre-TAVR use of VKA or DOAC**	89 (27.9)	84 (26.4)	0.7	101 (27.0)	86 (23.5)	0.3
**Cigarette use (current or former)**	83 (26.0)	86 (27.0)	0.8	137 (36.6)	139 (38.0)	0.8
**Chronic drug usage**	55 (17.2)	55 (17.3)	1.0	60 (16.0)	57 (15.6)	0.9
**Excessive alcohol use**	9 (2.8)	5 (1.6)	0.4	6 (1.6)	8 (2.2)	0.6
**Edoxaban initial dose[d]**						
30 mg	309 (96.9)	NA	NA	11 (2.9)	NA	NA
60 mg	9 (2.8)	NA	NA	361 (96.5)	NA	NA

Data are presented as n (%) unless otherwise noted. Categorical variables were presented as frequencies and percentages, and continuous variables were presented as means ± SD. Percentage calculations were based on the total number of patients in the analysis.

[a]Twenty patients were not treated in line with eDAC per locally approved label as defined for randomization (9 with eDAC at randomization received 60 mg as the first dose and 11 without eDAC at randomization received 30 mg as the first dose). This might be driven by changed eDAC by the time of first dose.

[b]Differences were tested using the unpaired Student's t-test or Fisher's exact test depending on the distribution of the variable.

[c]*P*-value for AF type is paroxysmal vs non-paroxysmal.

[d]Edoxaban initial dose was 0 mg in 2 patients from the no eDAC group where a >3-day treatment interruption was recorded and 0 mg in 1 patient from the eDAC group where a >3-day treatment interruption was recorded.

AF, atrial fibrillation; APT, antiplatelet therapy; BMI, body mass index; CABG, coronary bypass graft; CHA_2DS_2-VASc, Congestive heart failure, Hypertension, Age ≥75 (doubled), Diabetes, Stroke (doubled), Vascular disease, Age 65 to 74, and Sex category (female); CNS, central nervous system; COPD, chronic obstructive pulmonary disease; CrCl, creatinine clearance; DOAC, direct oral anticoagulant; eDAC, edoxaban dose adjustment criteria; HAS-BLED, Hypertension, Abnormal Renal and Liver Function, Stroke, Bleeding, Labile INR, Elderly, Drugs or Alcohol; INR, international normalized ratio; MI, myocardial infarction; PCI, percutaneous coronary intervention; PPI, proton pump inhibitor; SD, standard deviation; STS, Society of Thoracic Surgeons; TAVR, transcatheter aortic valve replacement; TIA, transient ischemic attack; VKA, vitamin K antagonist.

SUPPLEMENTAL TABLE 2. Antiplatelet Therapy Stratified by Patients With vs Without eDAC, Age, and Treatment Group During the Study

				Age <80 years			Age ≥80 years			eDAC			No eDAC		
**Parameter**	**eDAC[a]**	**No eDAC[a]**	***P*-value[b]**	**eDAC**	**No eDAC**	***P*-value[b]**	**eDAC**	**No eDAC**	***P*-value[b]**	**EDO**	**VKA**	***P*-value[b]**	**EDO**	**VKA**	***P*-value[b]**
N	637	740		98	262		539	478		319	318		374	366	
APT during the study[c], n (%)	347 (54.5)	435 (58.8)	0.1	57 (58.2)	156 (59.5)	0.8	290 (53.8)	279 (58.4)	0.1	175 (54.9)	172 (54.1)	0.9	216 (57.8)	219 (59.8)	0.6

[a]Patients were grouped by those with at least one eDAC or no eDAC as specified for randomization.

[b]*P*-values are from analysis of variance for numerical parameters and from Fisher's exact test for categorical parameters.

[c]APT therapy during the study was defined as exposure to at least one APT medication from first study drug dose to last study drug dose plus 3 days.

APT, antiplatelet; eDAC, edoxaban dose adjustment criteria; EDO, edoxaban; VKA, vitamin K antagonist.

SUPPLEMENTAL TABLE 3. Cause of Death in Patients With vs Without eDAC

Parameter	Overall (N = 1377)	eDAC (n = 637)	No eDAC (n = 740)
**Fatal Bleeding**	17 (1.2)	8 (1.3)	9 (1.2)
**Cause of Death, n (% of N)**	98 (7.1)	52 (8.2)	46 (6.2)
Cardiovascular, n (% of deaths)	57 (58.2)	33 (63.5)	24 (52.2)
Atherosclerotic vascular disease (excluding coronary)	1	0	1
Congestive heart failure/cardiogenic shock	15	10	5
Dysrhythmia	2	1	1
Intracranial hemorrhage	8	4	4
Non-intracranial hemorrhage	1	0	1
Other	8	4	4
Sudden or unwitnessed death	22	14	8
Malignancy, n (% of deaths)	4 (4.1)	2 (3.9)	2 (4.4)
Leukemia	1	1	0
Pancreatic	1	0	1
Skin	1	1	0
Stomach	1	0	1
Non-cardiovascular/Non-malignancy, n (% of deaths)	37 (37.8)	17 (32.7)	20 (43.5)
Accidental/trauma	1	0	1
Hepatobiliary	2	1	1
Infection	21	10	11
Other	11	5	6
Renal	1	1	0
Suicide	1	0	1

Data are presented as n (%) unless otherwise noted.

CABG, coronary bypass graft; eDAC, edoxaban dose adjustment criteria; PCI, percutaneous coronary intervention.

SUPPLEMENTAL FIGURE 1. Annualized On-Treatment Event Rates and Adjusted Competing Risk Analysis of Endpoints Comparing Patients With and Without eDAC

Endpoint	eDAC	n/N	Event rate (%/yr) (95% CI)	Adjusted hazard ratio[a,b] (95% CI)	*P*-value[a,b]
NACE[c]	Yes	127/637	17.00 (14.17, 20.22)	1.15 (0.871, 1.517)	0.3
	No	128/740	13.53 (11.29, 16.09)		
All-cause death	Yes	52/637	6.65 (4.97, 8.73)	1.38 (0.866, 2.206)	0.2
	No	46/740	4.66 (3.41, 6.21)		
CV death	Yes	33/637	4.22 (2.91, 5.93)	1.78 (0.948, 3.341)	0.07
	No	24/740	2.43 (1.56, 3.61)		
Non-CV death	Yes	19/637	2.43 (1.47, 3.80)	0.89 (0.446, 1.791)	0.8
	No	22/740	2.23 (1.40, 3.37)		
Ischemic stroke	Yes	20/637	2.58 (1.57, 3.98)	0.96 (0.492, 1.874)	0.9
	No	21/740	2.14 (1.32, 3.26)		
Major bleeding[c]	Yes	64/637	8.47 (6.53, 10.82)	0.94 (0.646, 1.377)	0.8
	No	75/740	7.86 (6.18, 9.85)		
Fatal major bleeding[c]	Yes	8/637	1.02 (0.44, 2.02)	1.03 (0.343, 3.084)	1.0
	No	9/740	0.91 (0.42, 1.73)		
ICH	Yes	15/637	1.93 (1.08, 3.18)	1.14 (0.537, 2.407)	0.7
	No	19/740	1.93 (1.16, 3.01)		
Major GI bleeding[c]	Yes	28/637	3.62 (2.41, 5.23)	0.66 (0.379, 1.165)	0.2
	No	44/740	4.59 (3.34, 6.16)		
Major non-GI bleeding[c]	Yes	40/637	5.26 (3.75, 7.16)	1.33 (0.804, 2.205)	0.3
	No	35/740	3.57 (2.48, 4.96)		
Death, stroke, SEE, and/or major bleeding	Yes	123/637	16.43 (13.66, 19.61)	1.14 (0.859, 1.516)	0.4
	No	123/740	12.97 (10.78, 15.48)		
Death, stroke, SEE, and/or life-threatening bleeding	Yes	86/637	11.17 (8.94, 13.80)	1.33 (0.933, 1.888)	0.1
	No	78/740	7.97 (6.30, 9.95)		
Death, persistent disability stroke, and/or life-threatening bleeding	Yes	66/637	8.51 (6.58, 10.83)	1.36 (0.911, 2.044)	0.1
	No	61/740	6.20 (4.74, 7.96)		

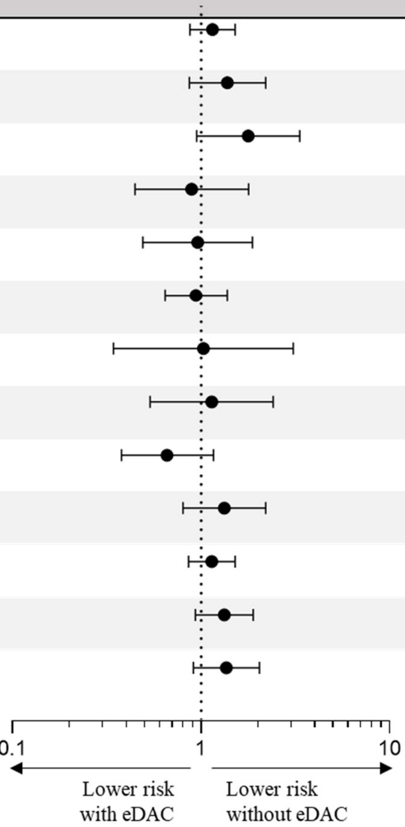

[a]Hazard ratios were only calculated for outcomes with ≥5 events in both groups.

[b]Fine and Gray regression models were adjusted for the competing risk of death and baseline differences including the following list of covariates: age, sex, hypertension, hypercholesterolemia, CABG, PCI within 30 days, GI disorder, cigarette use, and platelets. No imputation for missing data was done except binary variables where the 'No' category was assumed if not 'Yes.' All endpoints were adjusted for the competing risk of all-cause death, except CV death which was adjusted for the competing risk of non-CV death, and non-CV death which was adjusted for the competing risk of CV death. All-cause death and net clinical outcomes were not adjusted for any competing risk with results corresponding to the Cox regression analysis.

[c]The ISTH definition was used.

CABG, coronary artery bypass graft; CI, confidence interval; CV, cardiovascular; eDAC, edoxaban dose adjustment criteria; GI, gastrointestinal; ICH, intracranial hemorrhage; ISTH, International Society on Thrombosis and Haemostasis; NACE, net adverse clinical events; PCI, percutaneous coronary intervention; SEE, systemic embolic events.

SUPPLEMENTAL FIGURE 2. Annualized On-Treatment Event Rates and Adjusted Competing Risk Analysis of Endpoints Comparing Patients With and Without eDAC by Patients (A) Aged <80 Years or (B) Aged ≥80 Years

A) Patients aged <80 years

Endpoint	eDAC	n/N	Event rate (%/yr) (95% CI)	Adjusted hazard ratio[a,b] (95% CI)	*P*-value[a,b]
NACE[c]	Yes	20/98	17.29 (10.56, 26.70)	1.54 (0.852, 2.776)	0.2
	No	42/262	11.58 (8.34, 15.65)		
All-cause death	Yes	9/98	7.44 (3.40, 14.12)	2.23 (0.834, 5.950)	0.1
	No	13/262	3.41 (1.82, 5.84)		
CV death	Yes	7/98	5.79 (2.33, 11.92)	3.39 (0.948, 12.11)	0.06
	No	7/262	1.84 (0.74, 3.79)		
Non-CV death	Yes	2/98	1.65 (0.20, 5.97)		
	No	6/262	1.58 (0.58, 3.43)		
Ischemic stroke	Yes	3/98	2.50 (0.52, 7.31)		
	No	8/262	2.12 (0.91, 4.17)		
Major bleeding[c]	Yes	8/98	6.76 (2.92, 13.32)	1.12 (0.441, 2.819)	0.8
	No	23/262	6.23 (3.95, 9.35)		
Fatal major bleeding[c]	Yes	2/98	1.65 (0.20, 5.97)		
	No	2/262	0.53 (0.06, 1.90)		
ICH	Yes	2/98	1.65 (0.20, 5.97)		
	No	6/262	1.58 (0.58, 3.44)		
Major GI bleeding[c]	Yes	4/98	3.32 (0.91, 8.51)		
	No	13/262	3.51 (1.87, 6.00)		
Major non-GI bleeding[c]	Yes	5/98	4.20 (1.36, 9.80)	1.53 (0.495, 4.754)	0.5
	No	10/262	2.64 (1.27, 4.86)		
Death, stroke, SEE, and/or major bleeding	Yes	19/98	16.23 (9.77, 25.34)	1.62 (0.863, 3.047)	0.1
	No	38/262	10.38 (7.35, 14.25)		
Death, stroke, SEE, and/or life-threatening bleeding	Yes	14/98	11.76 (6.43, 19.72)	1.86 (0.905, 3.811)	0.09
	No	24/262	6.37 (4.08, 9.48)		
Death, persistent disability stroke, and/or life-threatening bleeding	Yes	10/98	8.31 (3.99, 15.29)	2.03 (0.860, 4.803)	0.1
	No	18/262	4.74 (2.81, 7.50)		

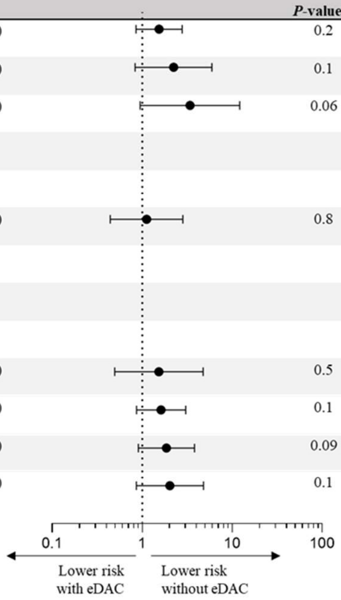

B) Patients aged ≥80 years

Endpoint	eDAC	n/N	Event rate (%/yr) (95% CI)	Adjusted hazard ratio[a,b] (95% CI)	*P*-value[a,b]
NACE[c]	Yes	107/539	16.95 (13.89, 20.48)	1.07 (0.783, 1.465)	0.7
	No	86/478	14.75 (11.80, 18.21)		
All-cause death	Yes	43/539	6.51 (4.71, 8.77)	1.19 (0.705, 1.997)	0.5
	No	33/478	5.43 (3.74, 7.63)		
CV death	Yes	26/539	3.94 (2.57, 5.77)	1.36 (0.658, 2.816)	0.4
	No	17/478	2.80 (1.63, 4.48)		
Non-CV death	Yes	17/539	2.58 (1.50, 4.13)	0.91 (0.433, 1.918)	0.8
	No	16/478	2.64 (1.51, 4.28)		
Ischemic stroke	Yes	17/539	2.59 (1.51, 4.15)	1.06 (0.493, 2.295)	0.9
	No	13/478	2.15 (1.14, 3.67)		
Major bleeding[c]	Yes	56/539	8.79 (6.64, 11.42)	0.92 (0.599, 1.408)	0.7
	No	52/478	8.88 (6.63, 11.65)		
Fatal major bleeding[c]	Yes	6/539	0.91 (0.33, 1.98)	0.64 (0.188, 2.203)	0.5
	No	7/478	1.15 (0.46, 2.37)		
ICH	Yes	13/539	1.98 (1.05, 3.39)	1.13 (0.464, 2.768)	0.8
	No	13/478	2.14 (1.14, 3.66)		
Major GI bleeding[c]	Yes	24/539	3.67 (2.35, 5.47)	0.56 (0.295, 1.066)	0.08
	No	31/478	5.27 (3.58, 7.49)		
Major non-GI bleeding[c]	Yes	35/539	5.45 (3.80, 7.58)	1.34 (0.772, 2.328)	0.3
	No	25/478	4.15 (2.69, 6.13)		
Death, stroke, SEE, and/or major bleeding	Yes	104/539	16.47 (13.46, 19.96)	1.06 (0.772, 1.450)	0.7
	No	85/478	14.60 (11.66, 18.05)		
Death, stroke, SEE, and/or life-threatening bleeding	Yes	72/539	11.07 (8.66, 13.94)	1.22 (0.821, 1.804)	0.3
	No	54/478	8.98 (6.74, 11.71)		
Death, persistent disability stroke, and/or life-threatening bleeding	Yes	56/539	8.55 (6.46, 11.10)	1.22 (0.776, 1.921)	0.4
	No	43/478	7.11 (5.15, 9.58)		

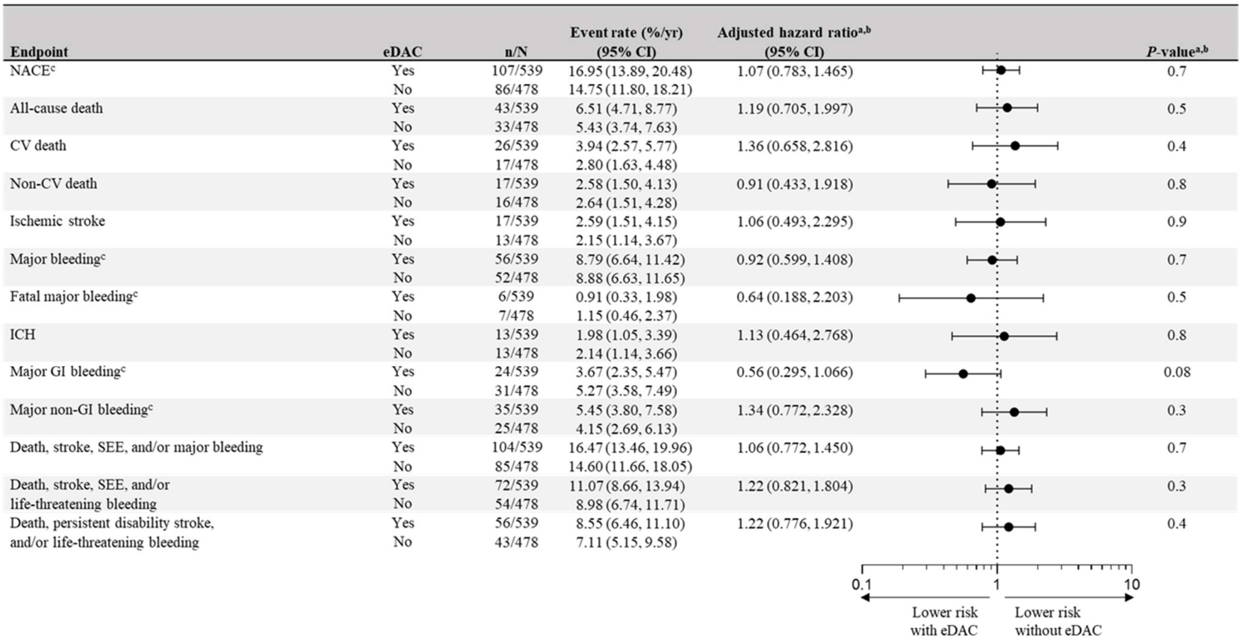

[a]Hazard ratios were only calculated for outcomes with ≥5 events in both groups.

[b]Fine and Gray regression models were adjusted for the competing risk of death and baseline differences including the following list of covariates: age, sex, hypertension, hypercholesterolemia, CABG, PCI within 30 days, GI disorder, cigarette use, and platelets. No imputation for missing data was done except binary variables where the 'No' category was assumed if not 'Yes.' All endpoints were adjusted for the competing risk of all-cause death, except CV death which was adjusted for the competing risk of non-CV death, and non-CV death which was adjusted for the competing risk of CV death. All-cause death and net clinical outcomes were not adjusted for any competing risk with results corresponding to the Cox regression analysis.

[c]The ISTH definition was used.

CABG, coronary artery bypass graft; CI, confidence interval; CV, cardiovascular; eDAC, edoxaban dose adjustment criteria; GI, gastrointestinal; ICH, intracranial hemorrhage; ISTH, International Society on Thrombosis and Haemostasis; NACE, net adverse clinical events; PCI, percutaneous coronary intervention; SEE, systemic embolic events.

Supplemental Figure 3. Annualized Event Rates and Competing Risk Analysis in the On-Treatment Study Period Comparing Patients With and Without eDAC by Treatment and Patients (A) Aged <80 Years or (B) Aged ≥80 Years

A) Patients aged <80 years

Endpoint	eDAC	Event rate, %/yr (n/N) Edoxaban	VKAs	Adjusted hazard ratio[a,b] (95% CI)	*P*-value[c]
NACE[d]	Yes	20.44 (13/52)	13.44 (7/46)	1.63 (0.64, 4.16)	
	No	10.68 (20/132)	12.54 (22/130)	0.85 (0.46, 1.56)	
	Overall	13.15 (33/184)	12.75 (29/176)	1.05 (0.64, 1.74)	0.2
All-cause death	Yes	7.45 (5/52)	7.43 (4/46)		
	No	4.13 (8/132)	2.67 (5/130)	1.55 (0.51, 4.75)	
	Overall	4.99 (13/184)	3.73 (9/176)	1.34 (0.57, 3.13)	0.6
CV death	Yes	5.96 (4/52)	5.57 (3/46)		
	No	2.07 (4/132)	1.60 (3/130)		
	Overall	3.07 (8/184)	2.49 (6/176)	1.23 (0.43, 3.51)	0.9
Non-CV death	Yes	1.49 (1/52)	1.86 (1/46)		
	No	2.06 (4/132)	1.07 (2/130)		
	Overall	1.92 (5/184)	1.25 (3/176)		0.6
Ischemic stroke	Yes	2.98 (2/52)	1.89 (1/46)		
	No	1.04 (2/132)	3.23 (6/130)		
	Overall	1.54 (4/184)	2.94 (7/176)		0.3
Major bleeding[d]	Yes	7.67 (5/52)	5.64 (3/46)		
	No	6.90 (13/132)	5.54 (10/130)	1.18 (0.51, 2.72)	
	Overall	7.10 (18/184)	5.56 (13/176)	1.26 (0.61, 2.57)	0.8
ICH	Yes	1.49 (1/52)	1.86 (1/46)		
	No	1.04 (2/132)	2.14 (4/130)		
	Overall	1.15 (3/184)	2.08 (5/176)		0.7
Major GI bleeding[d]	Yes	2.98 (2/52)	3.76 (2/46)		
	No	4.22 (8/132)	2.77 (5/130)	1.53 (0.50, 4.65)	
	Overall	3.89 (10/184)	2.99 (7/176)	1.33 (0.51, 3.47)	0.6
Major non-GI bleeding[d]	Yes	4.60 (3/52)	3.72 (2/46)		
	No	2.60 (5/132)	2.69 (5/130)	0.81 (0.22, 2.98)	
	Overall	3.10 (8/184)	2.92 (7/176)	0.98 (0.35, 2.80)	0.6

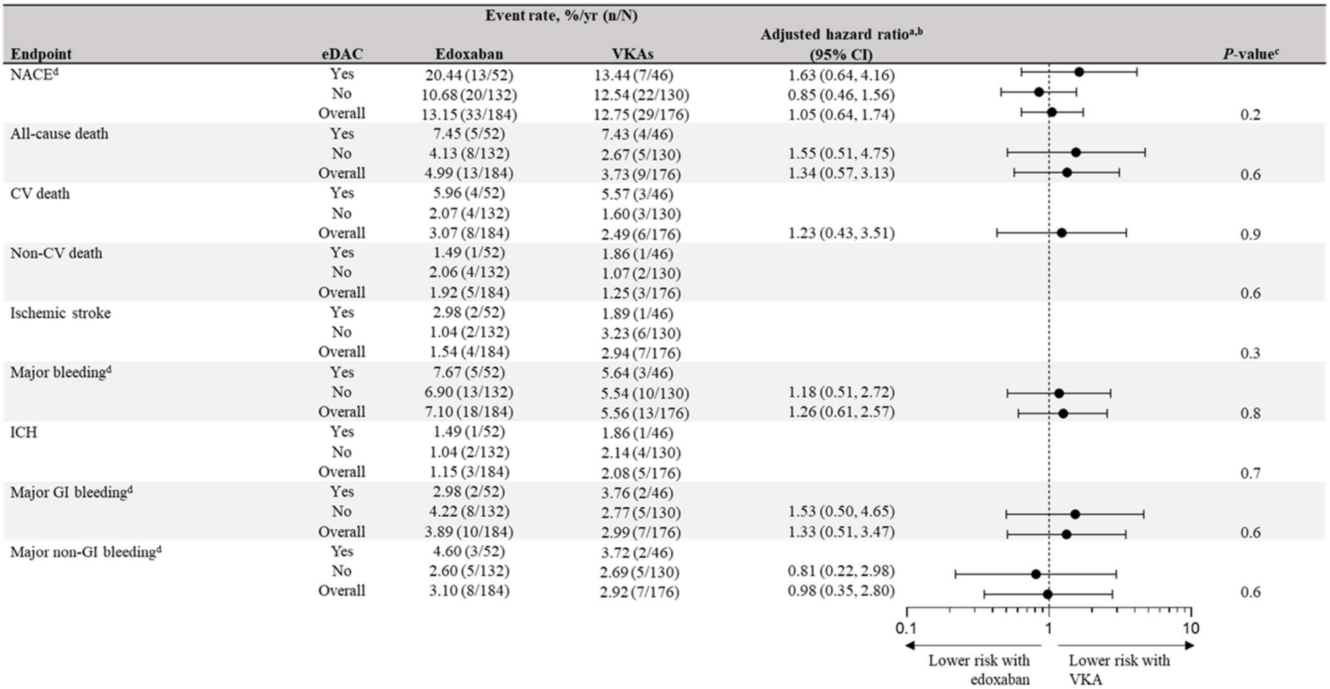

B) Patients aged ≥80 years

Endpoint	eDAC	Event rate, %/yr (n/N) Edoxaban	VKAs	Adjusted hazard ratio[a,b] (95% CI)	*P*-value[c]
NACE[d]	Yes	16.95 (56/267)	16.94 (51/272)	0.99 (0.67, 1.45)	
	No	16.89 (52/242)	12.35 (34/236)	1.38 (0.90, 2.13)	
	Overall	16.92 (108/509)	14.75 (85/508)	1.15 (0.86, 1.53)	0.3
All-cause death	Yes	6.03 (21/267)	7.04 (22/272)	0.85 (0.47, 1.55)	
	No	5.45 (18/242)	5.41 (15/236)	1.01 (0.51, 2.01)	
	Overall	5.75 (39/509)	6.28 (37/508)	0.92 (0.58, 1.44)	0.7
CV death	Yes	4.02 (14/267)	3.84 (12/272)	1.04 (0.48, 2.25)	
	No	3.03 (10/242)	2.52 (7/236)	1.20 (0.46, 3.13)	
	Overall	3.54 (24/509)	3.22 (19/508)	1.09 (0.60, 1.99)	0.8
Non-CV death	Yes	2.01 (7/267)	3.21 (10/272)	0.63 (0.24, 1.67)	
	No	2.42 (8/242)	2.89 (8/236)	0.84 (0.32, 2.21)	
	Overall	2.21 (15/509)	3.06 (18/508)	0.73 (0.37, 1.44)	0.7
Ischemic stroke	Yes	2.61 (9/267)	2.57 (8/272)	1.20 (0.45, 3.23)	
	No	1.83 (6/242)	2.53 (7/236)	0.74 (0.25, 2.23)	
	Overall	2.23 (15/509)	2.55 (15/508)	0.97 (0.47, 2.01)	0.5
Major bleeding[d]	Yes	9.56 (32/267)	7.94 (24/272)	1.17 (0.69, 2.00)	
	No	11.29 (35/242)	6.17 (17/236)	1.88 (1.05, 3.34)	
	Overall	10.40 (67/509)	7.10 (41/508)	1.47 (0.99, 2.17)	0.2
ICH	Yes	1.73 (6/267)	2.26 (7/272)	0.79 (0.26, 2.36)	
	No	1.52 (5/242)	2.89 (8/236)	0.53 (0.17, 1.59)	
	Overall	1.63 (11/509)	2.56 (15/508)	0.65 (0.30, 1.41)	0.6
Major GI bleeding[d]	Yes	4.65 (16/267)	2.59 (8/272)	1.75 (0.74, 4.12)	
	No	8.03 (25/242)	2.17 (6/236)	3.78 (1.55, 9.22)	
	Overall	6.25 (41/509)	2.39 (14/508)	2.63 (1.43, 4.84)	0.2
Major non-GI bleeding[d]	Yes	5.31 (18/267)	5.61 (17/272)	0.92 (0.47, 1.81)	
	No	3.66 (12/242)	4.73 (13/236)	0.80 (0.37, 1.73)	
	Overall	4.50 (30/509)	5.19 (30/508)	0.87 (0.52, 1.44)	0.8

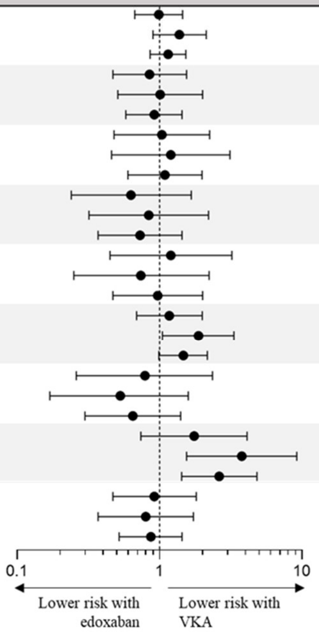

[a]Hazard ratios were only calculated for outcomes with ≥5 events in both groups.

[b]Fine and Gray regression models were adjusted for the competing risk of death. All endpoints were adjusted for the competing risk of all-cause death, except CV death which was adjusted for the competing risk of non-CV death, and non-CV death which was adjusted for the competing risk of CV death. All-cause death was not adjusted for any competing risk.

[c]*P*-value was based on a comparison of dose adjustment and treatment subgroups using a Fine and Gray model including dose adjustment and treatment subgroups and the interaction between the dose adjustment and treatment subgroups in the model. A significant interaction term indicates a differential dose adjustment subgroup effect within treatment subgroups.

[d]ISTH definition was used.

CI, confidence interval; CV, cardiovascular; eDAC, edoxaban dose adjustment criteria; GI, gastrointestinal; ICH, intracranial hemorrhage; ISTH, International Society on Thrombosis and Haemostasis; NACE, net adverse clinical events.
